# Cannabidiol (CBD) Use among children with juvenile idiopathic arthritis

**DOI:** 10.1186/s12969-021-00656-5

**Published:** 2021-12-13

**Authors:** Christopher J Failing, Kevin F. Boehnke, Meredith Riebschleger

**Affiliations:** 1 Department of Pediatric Rheumatology, C.S. Mott Children’s Hospital, Ann Arbor, United States MI; 2 Pediatric Rheumatology Department, Sanford Health, Fargo, United States ND; 3 University of Michigan, Ann Arbor, United States MI; 4 Anesthesiology Department, University of Michigan, Ann Arbor, United States MI

**Keywords:** Juvenile idiopathic arthritis, Cannabidiol, Pediatric rheumatology, Complementary and integrative medicine

## Abstract

**Background:**

Juvenile idiopathic arthritis (JIA) is common and difficult to treat. Cannabidiol (CBD) is now widely available, but no studies to date have investigated the use of CBD for JIA.

**Methods:**

We performed a chart review to identify patients with JIA at a Midwestern medical institution between 2017 and 2019. We surveyed primary caregivers of JIA patients using an anonymous, online survey with questions on caregiver knowledge and attitudes towards CBD. We compared respondents with no interest in CBD use vs. those contemplating or currently using CBD using descriptive statistics.

**Results:**

Of 900 reviewed charts, 422 met inclusion criteria. Of these, 236 consented to be sent a survey link, and *n*=136 (58%) completed surveys. Overall, 34.5% (*n*=47) of respondents reported no interest in using a CBD product for their child’s JIA, while 54% (*n*=79) reported contemplating using CBD and 7% (*n*=10) reported currently giving their child CBD. Only 2% of respondents contemplating or actively using a CBD product learned about CBD from their child’s rheumatologist, compared with television (70%) or a friend (50%). Most respondents had not talked to their child’s rheumatologist about using CBD. Of those currently using CBD, most used oral or topical products, and only 10% of respondents (*n*=1) knew what dose they were giving their child.

**Conclusions:**

Our results show infrequent use but a large interest in CBD among caregivers of children with JIA. Given CBD’s unknown safety profile in children with JIA, this study highlights a need for better studies and education around CBD for pediatric rheumatologists.

**Supplementary Information:**

The online version contains supplementary material available at 10.1186/s12969-021-00656-5.

## Background

Juvenile idiopathic arthritis (JIA) is the most common type of chronic arthritis in children, affecting 1 in 1000 children. It is an important cause of short and long-term disability and causes significant financial burden with annual direct medical costs ranging $400-$7,000 [1]. Effective treatments for JIA include non-steroidal anti-inflammatory drugs (NSAIDs), corticosteroids, disease modifying anti-rheumatic drugs (DMARDs), and biologic agents, but each carries potential adverse effects [2]. Indeed, parents and children frequently worry about side effects and the long-term safety of medications prescribed for JIA [3, 4]. As a result, many parents and children (34-92%) use complementary and integrative medicine (CIM) separately or in conjunction with standard treatment of JIA [5–10].

One such CIM treatment gaining popularity in the past years is cannabidiol (CBD), which is derived from *Cannabis sativa.* Since the removal of some CBD products from the Controlled Substances Act in 2018, a vast number of products made from hemp (*Cannabis sativa* with <0.3% Δ-9-tetrahydrocannabinol [THC]) have become available in brick and mortar retailers in topical, oral or inhaled forms [11]. CBD is non-intoxicating and has been widely advertised as a safe and natural therapy for many ailments including chronic pain, arthritis, other inflammatory diseases, and mental health conditions, resulting in frequent use for these conditions [12, 13]. In non-human animal studies, CBD reduces pain and inflammation due to arthritis, but these findings have not been validated in human studies [14, 15]. With the exception of Epidiolex, which is approved for the treatment of the rare seizure disorders Lennox Gastaut and Dravet Syndrome, CBD is minimally regulated by the Food and Drug Administration (FDA) [16, 17]. With the exception of these rare seizure disorders, evidence of a therapeutic benefit of CBD for pediatric conditions is lacking [18].

CBD’s safety profile has only been characterized among individuals with Dravet and Lennox Gastaut syndrome, so whether CBD is safe for use in healthy children or other pediatric populations remains unknown. Complicating matters, CBD is a promiscuous molecule that interacts with numerous systems in the body (e.g., serotonergic 5HT_1A_, endocannabinoid system as cannabinoid receptor 1 antagonist) [19, 20], and may interact with the metabolism of drugs commonly taken by children with JIA including prednisone and naproxen [21]. Further, testing of safety and potency of CBD products is not governed by a strong regulatory apparatus, [22, 23] and a recent *JAMA* study revealed that only 31% of CBD products sold online are accurately labeled for potency with 21% of samples containing THC [24]. As such, there are safety concerns about use in children, especially those with JIA.

As pediatric rheumatologists, the authors (C.F., M. R.) have been frequently asked about using CBD products to treat JIA symptoms, but to date, there is no literature available regarding the use of CBD in children with JIA. The objective of this study was to determine the frequency of CBD use among children with JIA and investigate caregiver knowledge and opinions about CBD use for their children.

## Methods

All study procedures and protocols were approved by the Institutional Review Board (IRB) at the University of Michigan (HUM00169198). We first conducted an administrative data query at the University of Michigan to identify all children ages 0-17 years of age at the time of a visit associated with the ICD-10 code for JIA between 1/1/2017 and 12/31/2019. That administrative data query identified 900 patients with ICD-10 codes for JIA.

### Participant eligibility

The charts of those 900 patients were then reviewed by C.F. Parents or guardians of patients were invited to participate in the study if the patient was younger than 18 years of age at the time of survey, had a diagnosis of JIA, had more than 1 visit to Pediatric Rheumatology clinic, and had been evaluated by a Pediatric Rheumatologist within the last 18 months. *N*=422 eligible participants were contacted by phone and invited to take an anonymous online survey created by the authors using a unique link through Qualtrics between December of 2019 and February of 2020. Only respondents interested in the survey were sent the unique link. Respondents were not compensated for completing the survey.

### Survey

The survey consisted of 83 items, some of which were variably displayed depending on participant’s responses. Questions addressed parent/guardian demographics (age, gender, ethnicity, education level, annual household income), use of complementary and integrative medicine (CIM) over lifetime (no use, 1 CIM, 2-4 CIMs, > 4 CIM), history of parent/guardian CBD product and cannabis use, child demographics and disease characteristics (age, gender, subtype of JIA, disease duration, parent/guardian report of disease activity at last rheumatology appointment, current rheumatologic medications used, co-morbid health conditions), and total number of CIM therapies used over child’s lifetime.

Respondents using CBD or contemplating CBD use for treatment of their child’s arthritis answered questions about sources of CBD information, perceptions of how CBD might improve their child’s arthritis, perceptions of the safety of CBD, and whether they had discussed CBD with their child’s provider. If respondents had not discussed CBD with their child’s healthcare provider, they were asked for the reasons why.

If parent/guardian reported using CBD product for child’s arthritis, they were asked questions about their CBD product(s), route of CBD administration, frequency of CBD use, parental perception of child’s disease activity pre and post-CBD use (using a 0-10 visual analogue scale), and total daily dosage of CBD (if known).

### Statistical analysis

We performed descriptive analyses, and present results as frequency, n (%) and mean +/- standard deviation for categorical and continuous variables, respectively. We used Fisher’s chi-square test to assess differences in categorical variables. Participants were divided into 2 comparison groups for analyses: currently using or contemplating starting a CBD product for their child and no interest in starting a CBD product. Participants using CBD for treatment of their child’s arthritis were not used for standalone comparison due to small sample size (*n*=10). All statistical analysis was performed using Microsoft Excel (2016, Microsoft Corporation).

## Results

### Participation

**Fig. 1:**
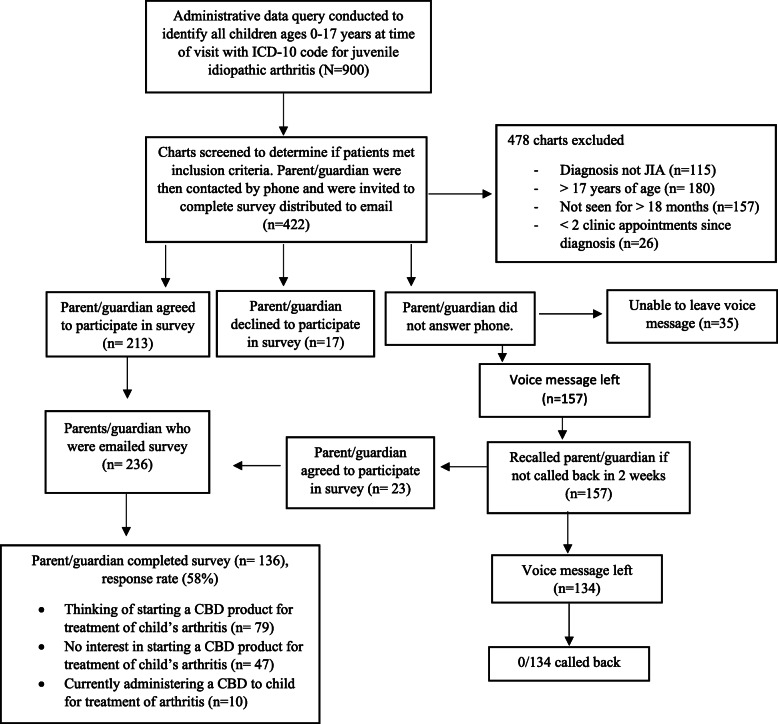
Flow diagram of study

Overall, 422 JIA patients met inclusion criteria. Of those, 236 parent/guardians agreed to be sent the survey link and 136 participants completed the survey (58% response rate, Fig. 1). 10 respondents (7%) reported using a CBD product to treat their child’s JIA, 79 respondents (58%) reported contemplating use of a CBD product to treat their child’s JIA, and 47 respondents (34.5%) reported no interest in starting a CBD product. Demographic characteristics of the survey respondents are shown in Table 1. The study population was largely white, had a bachelor’s degree or higher, and had an annual income of more than 50,000 dollars per year. A large majority of respondents in both groups reported using one or more CIM therapies in their lifetime. There was no significant difference in the specific types or number of CIM therapies used across groups. Report of high disease activity was more frequent among those currently using or contemplating CBD use than those not contemplating use.

**Table 1:** Correlation of demographics and disease characteristics

Parent/guardian and child demographics and disease characteristics	Total (*N*=136)	Not contemplating starting a CBD product for child (*N*=47)	Contemplating starting a CBD product for child (*N*=79) and using CBD product for child (*N*=10)	*P*-value
**Respondent parent/guardian age in years (mean +/- SD)**		29.7 (8)	26.5 (7)	
**Respondent Parent/guardian Gender- Female N (%)**	119 (87)	42 (89)	77 (86)	
**Race/ethnicity- N (%)**				
White/Caucasian	132 (97)	44 (94)	88 (98.9)	
Black/African American	2 (1)	1 (2)	1 (1.1)	
Asian American	2 (1)	2 (4)	0	
**Parent/guardian education level**				
High School or GED	20 (16)	2 (4)	18 (21)	χ2= 8.03 *p*=0.045*
Some college but no degree	26 (19)	7 (15)	19 (22)	
Associate degree	26 (19)	12 (26)	14 (16)	
Bachelor’s degree or higher	62 (46)	26 (55)	36 (41)	
**Income level- US dollars per year**				
Less than 19,000	3 (2)	1 (2)	2 (2)	χ2= 1.34 *p*= 0.72
20,000 to 49,000	27 (20)	7 (15)	20 (22)	
50,000 to 99,000	41 (30)	13 (28)	28 (32)	
100,000 or more	65 (48)	26 (55)	39 (44)	
**Child’s age in years- (mean +/- SD)**		11 (4)	11.9 (4)	
**Child gender: Female- N (%)**	95 (70)	31 (66)	64 (72)	
**JIA duration N (%)**				
< 6 months	2 (1)	2 (4)	0	χ2= 1.45 *p*= 0.69
6-12 months	9 (7)	3 (6)	6 (6)	
> 12- 24 months	20 (15)	7 (15)	13 (15)	
> 24 months	105 (77)	35 (75)	70 (79)	
**JIA Subtype N (%)**				
Oligoarticular	47 (35)	19 (47)	28 (32)	χ2= 7.93 *p*= 0.13
Polyarticular	53 (39)	12 (26)	41 (46)	
Psoriatic arthritis	6 (4)	3 (2)	3 (3)	
Systemic	14 (10)	8 (17)	6 (7)	
Ankylosing spondylitis	2 (1)	0	2 (2)	
Enthesitis related arthritis	1 (0.7)	1 (2)	0	
Unsure	12 (9)	3 (6)	9 (10)	
**Current disease activity N (%)**				
Active	59 (44)	15 (29)	44 (49)	χ2= 9.56 *p*= 0.022*
Inactive on medication	49 (36)	16 (34)	33 (37)	
Inactive off medication for < 1 year	18 (13)	11 (23)	7 (7.8)	
Clinical remission	10 (7)	5 (10)	5 (5.6)	
**Current medications reported using N (%)**				
None	22 (16)	10 (21)	12 (13)	χ2= 2.47 *p*= 0.48
NSAID	69 (51)	23 (49)	46 (51)	
Non-biologic DMARD	51 (38)	18 (38)	33 (37)	
Biologic DMARD	65 (48)	18 (38)	47 (52)	

^a^Column percentages are displayed

^b^P-values derived from the chi-squared (χ 2 ) or Wilcoxon tests

^c^DMARDs disease-modifying antirheumatic drugs, NSAIDs nonsteroidal anti-inflammatory drugs

^d^Medication categories are not mutually exclusive, therefore, medications do not sum to 100%

**Fig. 2:**
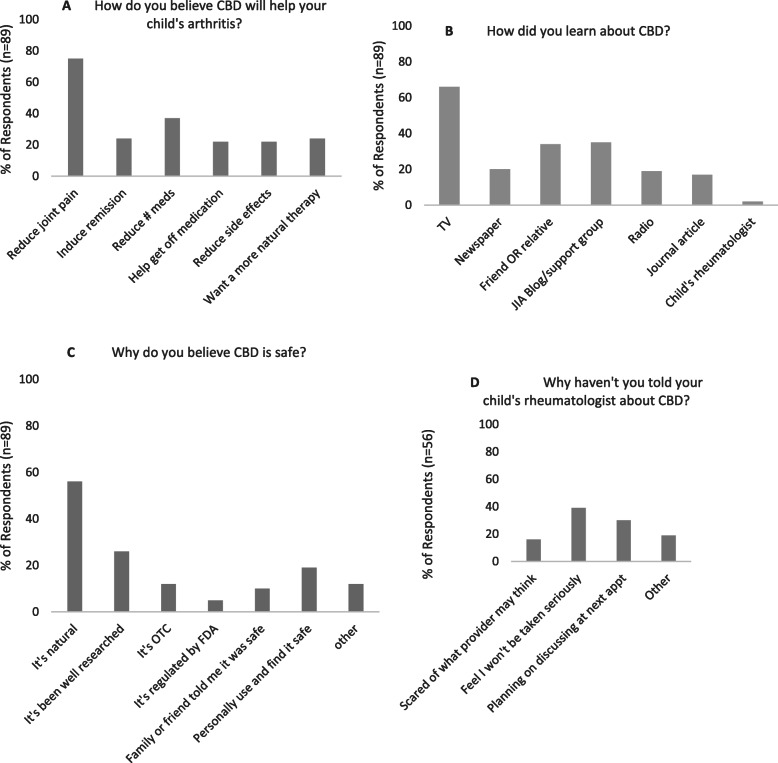
Parent/gaurdian perceptions of those using CBD for their child’s arthritis and those contemplating use of CBD (n=89) on how they percieve CBD will help their child’s arthritis (**A**), how they learned about CBD (**B**), perception of safety fo CBD (**C**). 56 respondents haven’t told their child’s rheumatologist for the following reasons (**D**)

A majority of those using CBD or contemplating using CBD for their child learned about it from TV (66%), a friend/relative (34%) or JIA online blog/support group (35%). Very few obtained information from a scientific journal article (17%) or their child’s rheumatologist (2%). Around half (52%) used 2 or more sources to learn about CBD. A majority of parents/guardians (75%) reported believing that CBD would reduce their child’s joint pain (Fig. 2 A), while only 15% of respondents reported believing that CBD has side effects. More than half of respondents reported thinking that CBD is safe because it is a natural product (Fig. 2 C). Nearly two-thirds (63%, *n* = 56) of respondents had not discussed using CBD with their child’s rheumatologist and over half (61%) of those did not plan on discussing with their child’s rheumatologist for the following reasons: scared of what provider may think (35%), felt they wouldn’t be taken seriously (29%), and believed rheumatologist would have no knowledge about CBD (18%).

### Contemplating CBD use

Respondents contemplating starting a CBD product for their child’s JIA (*n*=79) were interested in the following CBD products: CBD oil balm (30%), oil drops (25%), gummies (15%), soft gels/capsules (6.5%), and oil roll on (23%). Around a third (32%) of respondents were unsure what products they were interested in. Of those respondents (*n*=52) who were interested in starting a CBD product, 32.6% were interested in only oral CBD, 36.5% in a combination of oral and topical CBD, and 30.7% were interested only in topical CBD.

### Current CBD use

Respondents using CBD products for their child’s JIA (*n*=10) reported administering CBD orally (50%) or topically (50%). The majority (60%) reported using CBD on an as needed basis, while 40% reported using CBD on a scheduled basis. Overall, 40% reported administering CBD once per day, 20% twice per day and 40% at least three times per day. Respondents who reported administering CBD as needed (*n*=6) gave it for joint pain (66%), joint swelling (50%), joint stiffness (66%), and/or when their child requested it (33%). Respondents reported their child’s overall wellbeing to be an average 3.6 prior to starting CBD (0 = very poor, 10 = very good) and 5.3 after taking a CBD product. Half (50%, *n*=5) of parents reported improvement of their child’s wellbeing after they started CBD while 30% reported no change in their child’s wellbeing and 20% reported decreased well-being. Respondents used the following CBD products: oil drops (40%), lotion (10%), soft gels (10%) and oil balm (40%). Only one respondent knew the total dose of CBD administered per day (20 mg daily) while 70% (*n*=7) were unsure and 20% (*n*=2) reported they believed that the dose of CBD was irrelevant.

## Discussion

To our knowledge, this is the first study exploring parent/guardian knowledge and opinions regarding CBD use for their children with JIA. We found that while CBD use is infrequent, there is a strong parent/guardian interest in using CBD for treating JIA, especially among respondents reporting more active disease and a longer disease course. Use of stronger medications such as biologics, on the other hand, was not associated with a significant difference in CBD interest. These findings are consistent with other studies showing that children with JIA use CIM more frequently if they have more active disease and longer disease duration, and that use of immunosuppressive or biologic medications is not a factor related to CIM use among children with JIA [6, 25].

The majority of the survey respondents learned about CBD from television, the internet (JIA online blog/support group), or friend or family member with only a small percentage of respondents learned from a health care provider or scientific study, mirroring results from other studies of adults using CBD oil or cannabis [26, 27]. Our study further showed that many parent/guardians are not discussing CBD with their child’s rheumatologist. This is because they expressed worry that their child’s rheumatologist would negatively judge them and or not take them seriously if they discussed their experience with or interest in CBD. This finding is similar to a recent study in which only 9.6% of young adults reported discussing CBD usage with their healthcare provider [27]. Previous studies evaluating CIM use in adolescents with JIA have demonstrated similarly low rates of discussions with their health care provider, [10] and parents of children with other chronic health conditions have reported similar reasons for not discussing CIM with their child’s health provider. These results suggest that providers need CBD and CIM-related education to better serve individuals with JIA, and also that providers need to specifically ask about use of CBD and other CIM modalities.

As CBD becomes increasingly more popular, parental interest in using CBD to treat their child’s health conditions continues to grow. The use of the search terms for “CBD for children” and “CBD for kids” have increased since 2018, [22] and numerous blog posts and other forms of media report positive results from giving CBD to children [22]. These forms of media do often mention preclinical CBD research conducted in mice, which demonstrate that CBD has potent anti-inflammatory and analgesic effects in induced inflammatory arthritis [14, 15]. Further, some small clinical trials of CBD in adults do show that CBD may have analgesic activity (in neuropathy and temporomandibular joint disorder [28, 29]) and short-term anxiolytic effects, [30–32] and several clinical trials of CBD in arthritis are ongoing (for example, in rheumatoid arthritis) [33]. However, what is often not communicated is that studies on safety and efficacy of CBD among children with those symptoms (e.g., pain, inflammation) have not yet been conducted. As such, additional rigorous research is needed to investigate whether these preliminary therapeutic findings translate to the JIA context.

Consistent with prior reports about CBD administration among young adults, [12, 27] a majority of those using CBD for their child’s arthritis are administering CBD orally (60%) on an as needed basis as often as several times per day for joint pain and/or stiffness. In addition, 69% of those contemplating CBD expressed interest in an oral CBD product (alone or in combination with topical CBD). This strong interest in oral CBD is important to note, as CBD has been suggested to interact with the liver enzyme cytochrome P450 and could interfere with the metabolism of several commonly prescribed rheumatologic medications, including prednisone, naproxen, and tofacitinib, potentially leading to increased drug levels and increased risk of toxicity.

The large majority of respondents believed CBD is safe because it is a natural product and did not believe there were adverse effects of CBD. Surprisingly, only 1 of 10 participants currently giving their child CBD knew what dose they were administering. The overall safety of CBD for healthy children or other clinical populations remains unknown but the Epidiolex trials, [16, 34] which used high doses of CBD, reported non-serious adverse effects in children including dry mouth, sedation, and/or decreased appetite. Other studies have reported similar adverse effects in young adults or adults taking CBD [13, 27].

## Limitations

Respondents of both panels were similar in terms of race/ethnicity; education, age, and gender, however, > 95% of respondents were white/Caucasian which is not representative of the JIA patient population at our institution or in the US. Survey links were only generated for parents or guardians who expressed interest in participating in the study, so selection bias was likely present. In addition, respondents may have interpreted survey questions differently than we intended and wording of questions may have introduced bias. Finally, we only queried the parents/guardians of individuals with JIA rather than directly asking individuals with JIA about their experiences with or interest in CBD.

## Conclusions

As CBD continues to gain popularity, parental interest in CBD for treating their child’s health condition(s) will likely increase. In this study, we show that while CBD use is currently infrequent for JIA, many parents/guardians are interested in using CBD to help with JIA symptoms. As such, is important that pediatric rheumatologists and other pediatric providers educate themselves about CBD to increase their comfort in discussing CBD and its potential safety issues with their patients and/or parents. Such efforts should focus on harm-reduction, communicating uncertainty without harming the patient-physician relationship, and guiding interested parties to reliable sources on CBD (e.g. the Arthritis Foundation)[35] to ensure that they are obtaining information based on scientific evidence. In addition, rigorous clinical studies are warranted to investigate both safety and efficacy of CBD in JIA to bridge the gap in knowledge.

## Supplementary information

**Additional file 1**

## Data Availability

The datasets used and analyzed during the current study are available from the corresponding author on reasonable request.

## Abbreviations

CBD: Cannabidiol
JIA: juvenile idiopathic arthritis
NSAID: Non-steroidal anti-inflammatory drugs
DMARDs: Disease modifying anti-rheumatic drugs
CIM: Complementary and integrative medicine
THC: Tetrahydrocannabinol
FDA: Food and Drug Administration
IRB: Institutional Review Board
