# Supplementary material for: Cannabidiol (CBD) Use among children with juvenile idiopathic arthritis
Source: Pediatr Rheumatol Online J. 2021 Dec 13;19:171. doi: 10.1186/s12969-021-00656-5 (PMC8670290; doi:10.1186/s12969-021-00656-5)
Supplement: Supplementary file 1 — Additional file 1 [file 12969_2021_656_MOESM1_ESM.pdf]

The use of cannabidiol (CBD) in patients with juvenile idiopathic arthritis

Start of Block: Opening

Q1 The purpose of this study is to understand the use of cannabidiol (CBD) in children with juvenile idiopathic arthritis. This is a stand-alone survey. All data are anonymous. Any public presentations or publications using these data will be in aggregate; for example, the percent of respondents who used CBD oil.<div>
</div><div>We ask this survey be completed by the child's adult family member who is most responsible for giving their medications. The minimum age for the survey respondent is 18 years or older. </div><div>
</div><div>If you have more than one child with JIA please answer questions for only one child (your choice). This survey should take approximately 10-15 minutes to complete. </div><div>
</div><div>By continuing to complete this survey, you agree to have the information that you provide used for research purposes by the identified investigators at the University of Michigan. You are free to stop the survey at any time, including now.</div><div>
</div><div>The information you provide will help inform physicians, researchers, policy-makers, and patients about use of CBD in children with juvenile arthritis. </div><div>
</div><div>Please direct any questions or concerns you may have about this survey to umhs-peds-rheu@med.umich.edu</div><div>
</div><div>Investigators:</div><div>
</div><div>Christopher Failing, MD</div><div>
</div><div>Meredith Riebschleger, MD</div>

End of Block: Opening

Start of Block: Parent demographics

Q2 How old are you (in years)?

- ○ 18 (4)
- ○ 19 (5)
- ○ 20 (6)
- ○ 21 (7)
- ○ 22 (8)
- ○ 23 (9)
- ○ 24 (10)
- ○ 25 (11)
- ○ 26 (12)
- ○ 27 (13)
- ○ 28 (14)
- ○ 29 (15)
- ○ 30 (16)
- ○ 31 (17)
- ○ 32 (18)
- ○ 33 (19)
- ○ 34 (20)
- ○ 35 (21)
- ○ 36 (22)
- ○ 37 (23)
- ○ 38 (24)

- ○ 39 (25)
- ○ 40 (26)
- ○ 41 (27)
- ○ 42 (28)
- ○ 43 (29)
- ○ 44 (30)
- ○ 45 (31)
- ○ 46 (32)
- ○ 47 (33)
- ○ 48 (34)
- ○ 49 (35)
- ○ 50 (36)
- ○ 51 (37)
- ○ 52 (38)
- ○ 53 (39)
- ○ 54 (40)
- ○ 55 (41)
- ○ 56 (42)
- ○ 57 (43)
- ○ 58 (44)
- ○ 59 (45)

- ○ 60 (46)
- ○ 61 (47)
- ○ 62 (48)
- ○ 63 (49)
- ○ 64 (50)
- ○ 65 (51)
- ○ 66 (52)
- ○ 67 (53)
- ○ 68 (54)
- ○ 69 (55)
- ○ 70 (56)
- ○ 71 (57)
- ○ 72 (58)
- ○ 73 (59)
- ○ 74 (60)
- ○ 75 (61)
- ○ 76 (62)
- ○ 77 (63)
- ○ 78 (64)
- ○ 79 (65)
- ○ 80 (66)

- 81 (67)
- 82 (68)
- 83 (69)
- 84 (70)
- 85 (71)
- 86 (72)
- 87 (73)
- 88 (74)
- 89 (75)
- 90 (76)
- 91 (77)
- 92 (78)
- 93 (79)
- 94 (80)
- 95 (81)
- 96 (82)
- 97 (83)
- 98 (84)
- 99 (85)
- 100 (86)
- Prefer not to answer (87)

Q3 What gender are you?

- Male (1)
- Female (2)
- Transgender (gender non-conforming) (3)

Q4 What is your relationship to the child?

- Mother (1)
- Father (2)
- Step-mother (3)
- Step-father (4)
- Grandparent (5)
- Aunt (6)
- Uncle (7)
- Foster parent (8)
- Other family member (9)

Q5 What is the highest grade or level of school you have completed or the highest degree you have received?

- Less than high school (1)
- High school graduate (2)
- GED or equivalent (3)
- Some college, no degree (4)
- Associate degree; occupational, technical, or vocational program (5)
- Associate degree: academic program (6)
- Bachelor's degree (example BA, BS, AB, BBA) (7)
- Master's degree (example: MA, MS, MBA, MEng, Med) (8)
- Professional degree (example: MD, DDS, DVM, JD, DPT) (9)
- Doctoral degree (PhD) (10)

Q6 What category best describes your ethnicity? One or more categories may be marked.

- American Indian/Alaskan Native (1)
- Asian (2)
- Black of African American (3)
- Native Hawaiian/Pacific Islander (4)
- White/Caucasian (5)
- Other (6)
- Prefer not to answer (7)

Q7 What is your annual household income (in $US)?

- Less than 20,000 per year (1)
- 20,000 to 49,000 per year (2)
- 50,000 to 99,000 per year (3)
- 100,000 or more (4)

Q8 Are you currently using any of the following types of therapies, or have you used them in the past? Choose all that apply

- ▢ Massage therapy (1)
- ▢ Energy Work (2)
- ▢ Yoga (3)
- ▢ Acupuncture (4)
- ▢ Herbal supplements (5)
- ▢ Tai Chi (6)
- ▢ Spiritual healing (7)
- ▢ Chiropractic therapy (8)
- ▢ Detoxification therapy (9)
- ▢ Reiki healing (10)
- ▢ Essential oils (11)
- ▢ Cupping therapy (12)
- ▢ Aromatherapy (13)
- ▢ Reflexology (14)
- ▢ Crystal healing (15)
- ▢ Hydrotherapy (16)
- ▢ Hypnosis (17)
- ▢ Magnetic therapy (18)
- ▢ Aqua therpapy (19)

- [ ] Physical therapy/occupational therapy (20)
- [ ] Other (21)
- [ ] I have never used any of the therapies listed above (22)

Q9 If you selected "other" in the previous question, please type in any other therapies you have used in the past or are currently using.

____

End of Block: Parent demographics

Start of Block: Parental CBD oil use

Q11 Have you taken CBD in the past or are currently using?

- ○ I have never used CBD (1)
- ○ Not taking currently but have taken in the past (2)
- ○ Currently using (3)

Q12 If you have never used CBD , are there any family members living with your child who are using CBD ?

- ○ yes (1)
- ○ No (2)

Q13 For what medical condition or symptom do you use or have used CBD in the past for? Select all options that apply

- ▢ Acute pain (1)
- ▢ Rheumatoid arthritis (2)
- ▢ Anxiety (3)
- ▢ Back pain (4)
- ▢ Cancer (5)
- ▢ Inflammatory bowel disease (6)
- ▢ Fibromyalgia (7)
- ▢ Migraine (8)
- ▢ Nausea (9)
- ▢ other (10)

Q14 If you answered "other" in the previous question, please type in the name of the medical condition

____

Q16 How has CBD helped the medical condition for which you are using it for? (0= did not help, 10= helped alot) Move circle to number that corresponds on how CBD has helped you.

	0	1	2	3	4	5	6	7	8	9	10
Click to write Choice 1 ()											

Q17 Have you ever or in the past year used cannabis?

- ○ Recreational use only (1)
- ○ Medically only (2)
- ○ A combination of recreationally and medically (3)
- ○ Have not used (4)

End of Block: Parental CBD oil use

Start of Block: Child demographics and CBD use

Q18 How old is your child (in years)?

- Less than 1 (1)
- 1 (2)
- 2 (3)
- 3 (4)
- 4 (5)
- 5 (6)
- 6 (7)
- 7 (8)
- 8 (9)
- 9 (10)
- 10 (11)
- 11 (12)
- 12 (13)
- 13 (14)
- 14 (15)
- 15 (16)
- 16 (17)
- 17 (18)
- 18 (19)
- 19 (20)
- 20 (21)

- 21 (22)
- 22 (23)
- 23 (24)
- 24 (25)
- 25 (26)

Q19 What is your child's gender?

- Male (1)
- Female (2)
- Transgender (gender non-conforming) (3)

Q20 What type of juvenile arthritis (JIA) does your child have?

- Oligoarticular JIA (1)
- Polyarticular JIA (2)
- Enthesitis related arthritis (ERA) (3)
- Psoriatic arthritis (4)
- Systemic JIA (5)
- Ankylosing spondylitis (6)
- Unsure (7)

Q21 Approximately how long has your child had juvenile idiopathic arthritis?

- Less than 6 months (1)
- 6 to 12 months (2)
- 12 to 24 months (3)
- More than 24 months (4)

Q22 At my child's last appointment with his or her rheumatologist, their arthritis was:

- Active (1)
- In remission (also called inactive) but still on medications (2)
- In remission (also called inactive) and off medications for less than a year (3)
- In clinical remission (off all medications for > 1 year) (4)
- Unsure (5)

Q23 Current medication(s) prescribed by your child's rheumatologist. Choose all that apply

- [ ] NSAID (naproxen, meloxicam (mobic), sulindac, indomethacin, oxaprozin (Daypro), Celebrex or Relafen) (1)
- [ ] Injectable methotrexate (2)
- [ ] Oral methotrexate (3)
- [ ] Sulfasalazine (4)
- [ ] Leflunomide (Arava) (5)
- [ ] Hydroxychloroquine (Plaquenil) (6)
- [ ] Anakinra (Kineret) (7)
- [ ] Tocilizumab (Actemra) (8)
- [ ] Adalimumab (Humira) (9)
- [ ] Entanercept (Enbrel) (10)
- [ ] Infliximab (Remicade) (11)
- [ ] Secukinumab (Cosentyx) (12)
- [ ] Tofacitinib (Xeljanz) (13)
- [ ] Ustekinumab (Stelera) (14)
- [ ] Rituximab (Rituxan) (15)
- [ ] Golimumab (Simponi) (16)
- [ ] Other (17)

Q24 If you answered "other" in the previous question, please list any other medication(s) prescribed by your child's rheumatologist to treat their arthritis

____

Q25 Does you child have any of the following health conditions? Choose all that apply

- ☐ Amplified musculoskeletal pain syndrome (AMPS) (1)
- ☐ Macrophage activation syndrome (MAS) (2)
- ☐ Uveitis (3)
- ☐ Seizure disorder (4)
- ☐ ADHD (5)
- ☐ Depression (6)
- ☐ Anxiety (7)
- ☐ Chronic fatigue (8)
- ☐ Irritable bowel syndrome (9)
- ☐ Functional abdominal pain (10)
- ☐ POTS (11)
- ☐ Migraine headaches (12)
- ☐ Sleep disorder (13)
- ☐ None of the conditions listed above (14)

Q26 Is your child currently using any of the following types of therapies? Choose all that apply

- ☐ Massage therapy (1)
- ☐ Yoga (2)
- ☐ Energy work (3)
- ☐ Acupuncture (4)
- ☐ Herbal supplements (5)
- ☐ Tai Chi (6)
- ☐ Spritual healing (7)
- ☐ Chiropractic therapy (8)
- ☐ Detoxification therapy (9)
- ☐ Reiki healing (10)
- ☐ Essential oils (11)
- ☐ Cupping therapy (12)
- ☐ Aromatherapy (13)
- ☐ Bio-field therapy (14)
- ☐ Crystal healing (15)
- ☐ Reflexology (16)
- ☐ Hydrotherapy (17)
- ☐ Hypnosis (18)
- ☐ Magnetic therapy (19)
- ☐ Aqua therapy (20)

- [ ] Physical therapy/occupational therapy (21)
- [ ] My child is not using any of the therapies listed above (22)
- [ ] other (23)

Q27 If you selected "other" in the previous question, please type in what other therapies your child is currently using.

____

Q29 Has your child previously used any of the following types of therapies? Choose all that apply

- Massage therapy (1)
- Yoga (2)
- Energy work (3)
- Acupuncture (4)
- Herbal supplements (5)
- Tai Chi (6)
- Spritual healing (7)
- Chiropractic therapy (8)
- Detoxification therapy (9)
- Reiki healing (10)
- Essential oils (11)
- Cupping therapy (12)
- Aromatherapy (13)
- Bio-field therapy (14)
- Crystal healing (15)
- Reflexology (16)
- Hydrotherapy (17)
- Hypnosis (18)
- Magnetic therapy (19)

- [ ] Aqua therapy (20)
- [ ] Physical therapy/occupational therapy (21)
- [ ] My child has not previously used any of the therapies listed above (22)
- [ ] other (23)

Q81 If you selected "other" in the previous question, please type in what other therapies your child previously used.

____

Q28 Is your child on any dietary restriction that is being used to treat their arthritis? Choose all that apply

- [ ] Not on any dietary restriction (1)
- [ ] Gluten free (2)
- [ ] Low sugar diet (3)
- [ ] Dairy free (4)
- [ ] Vegan (5)
- [ ] Vegetarian (6)
- [ ] Low FODMAP diet (7)
- [ ] Anti-inflammatory diet (8)
- [ ] PALEO diet (9)
- [ ] Other (10)

Q30 If you selected "other" in the previous question, please type in what other dietary restriction your child is using to treat their arthritis

____

Q31 Is your child using a CBD product for treatment of their arthritis?

- Yes (1)
- No (2)

Q32 Have you thought about using a CBD product to treat your child's arthritis?

- Yes (1)
- No (2)
- Maybe (3)

Q33 Is your child taking CBD by mouth or applied to skin?

- By mouth only (1)
- Applied to skin (2)
- Both by mouth and applied to skin (3)

Q62 What type of CBD product is your child using? Select all that apply

- CBD oil balm (1)
- CBD oil spray (2)
- CBD drops (3)
- CBD soft gels or capsules (4)
- CBD gummies (5)
- CBD oil roll ons (6)
- CBD tincture powder (7)
- CBD isolate (8)
- Other (9)

Q82 If you selected "other" in the previous question, please type in what other CBD products is your child is using.

____

Q34 How long has your child been using CBD?

- Less than 6 months (1)
- 6 to 12 months (2)
- More than 12 months (3)

Q36 On average, how many times a day does your child use CBD?

- ○ Less than once daily (1)
- ○ Once (2)
- ○ Twice (3)
- ○ Three times (4)
- ○ Four times (5)
- ○ Five or more times (6)

Q35 Is your child using CBD on a scheduled basis (example everyday) or as needed?

- ○ Scheduled basis (1)
- ○ As needed (2)
- ○ Scheduled and as needed (3)

Q37 If using CBD as needed, when do you decide to give your child CBD? Choose all that app

- ☐ Joint pain (1)
- ☐ Joint swelling (2)
- ☐ Joint stiffness (3)
- ☐ Difficulty walking (4)
- ☐ When they're having side effects from their other medications (5)
- ☐ When my child requests for it (6)
- ☐ Other (7)

Q38 If you selected "other" in the previous question, please type in the reason(s) that make you decide to give CBD to your child

____

Q39 How did you learn about CBD? Choose all that apply

- ▢ TV (1)
- ▢ Newspaper (2)
- ▢ Friend or relative (3)
- ▢ JIA online support group (4)
- ▢ JIA blog (5)
- ▢ Radio (6)
- ▢ My own internet research (7)
- ▢ From by child's rheumatologist (8)
- ▢ From another physicain (9)
- ▢ Scientific journal article (10)
- ▢ Arthritis foundation (11)
- ▢ Other (12)

Q40 If you selected "other" in the previous question, please type in other ways you learned about CBD

____

Q41 How much research did you perform prior to starting CBD for your child?

- ○ A great deal (1)
- ○ A lot (2)
- ○ A moderate amount (3)
- ○ A little (4)
- ○ None at all (5)

Q42 What sources from your research did you find most credible? Choose all that apply

- ▢ TV (1)
- ▢ Newspaper (2)
- ▢ Radio (3)
- ▢ Friend or relative (4)
- ▢ Internet website (5)
- ▢ JIA online support group (6)
- ▢ JIA blog (7)
- ▢ Arthritis foundation (8)
- ▢ Scientific journal article (9)
- ▢ My child's rheumatologist (10)
- ▢ Another physican (11)
- ▢ Other (12)

Q43 If you selected "other" in the previous question, please type in what other sources from your research did you find most credible

____

Q44 How do you believe CBD will help your child's arthritis? Choose all that apply

- ▢ Reduce joint pain and inflammation (1)
- ▢ Keep or get arthritis into remission (2)
- ▢ Help reduce number of other medications prescribed (3)
- ▢ Help my child get off medications prescribed by their rheumatologist (4)
- ▢ Help reduce side effects of other medications (5)
- ▢ A natural therapy is effective for treating arthritis (6)
- ▢ I'm personally using a CBD product and found it to be helpful (7)
- ▢ Other (8)

Q45 If you selected "other" in the previous question, please type in other ways you believe CBD will help your child's arthritis

____

Q46 Over the past week, how many days did you worry about the medications your child is using for treatment of their arthritis?

- ○ 0 (1)
- ○ 1 (2)
- ○ 2 (3)
- ○ 3 (4)
- ○ 4 (5)
- ○ 5 (6)
- ○ 6 (7)
- ○ 7 (8)

Q47 Over the past week, how many days did your child have side effects from the medications prescribed by their rheumatologist?

- ○ 0 (1)
- ○ 1 (2)
- ○ 2 (3)
- ○ 3 (4)
- ○ 4 (5)
- ○ 5 (6)
- ○ 6 (7)
- ○ 7 (8)

Q48 Do you think that there are side effects of CBD?

- yes (1)
- Maybe (2)
- No (3)

Q49 How safe or unsafe do you think CBD is?

- Very unsafe (1)
- Unsafe (2)
- Safe (3)
- Very safe (4)

Q50 Why do you believe CBD oil is safe? Choose all that apply

- It's natural (1)
- It's available over the counter (2)
- Lots of research on CBD has been done (3)
- It's regulated by the Food and Drug Administration (FDA) (4)
- Friend or family member told me it was safe (5)
- Personal experience with CBD oil (6)
- Family of another child with JIA told me it was safe (7)
- Other (8)

Q51 If you selected "other' in the previous question, please type in other reasons why you think CBD is safe

____

Q52 How was your child's arthritis prior to starting CBD ? (0= very poor, 10= very good). Move circle to number you've selected

	0	1	2	3	4	5	6	7	8	9	10
Click to write Choice 1 ()											

Q54 How was your child's arthritis since starting CBD ? (0=very poor, 10= very good). Move circle to number you've selected

	0	1	2	3	4	5	6	7	8	9	10
Click to write Choice 1 ()											

Q55 Have you talked about CBD with your child's rheumatologist?

- Yes (1)
- No (2)

Q59 If you selected "other" in the previous question, please type in other reason(s) you haven't told your child's rheumatologist about CBD.

____

Q56 How did your child's rheumatologist react when you told him or her?

- ○ Seemed upset (1)
- ○ Neutral (2)
- ○ Seemed interested (3)
- ○ Seemed uninterested (4)
- ○ Other (5)

Q57 If you selected "other" in the previous question, how else did your child's rheumatologist react when you told him or her about CBD

____

Q58 Why have you not told your child's rheumatologist about starting CBD? Choose all that apply

- ▢ Scared of what provider may think (1)
- ▢ Feel I won't be taken seriously (2)
- ▢ Haven't had the chance to talk to rheumatologist yet but planning to at next appointment (3)
- ▢ I don't think my child's rheumatologist will know anything about CBD oil (4)
- ▢ Other (5)

Q60 Have you stopped a medication prescribed by your child's arthritis since starting CBD?

- Yes (1)
- No (2)

Q61 Have you told you child's rheumatologist about stopping a medication they prescribed?

- Yes (1)
- No (2)
- Planning to but have not yet had the chance (3)

Q63 Do you know what dose of CBD your child is taking?

- Yes (1)
- No (2)
- Dose doesn't matter (3)

Q64 If you answered "yes" in the previous question, please type in the dose of CBD your child is using

____

Q65 On an average month, how much money do you spend on CBD oil products for your child?

- Less than 20 dollars (1)
- 20 to 39 dollars (2)
- 40 to 59 dollars (3)
- 60 to 79 dollars (4)
- 80 to 99 dollars (5)
- 100 to 199 dollars (6)
- 200 to 299 dollars (7)
- 300 to 399 dollars (8)
- 400 to 499 dollars (9)
- More than 500 dollars (10)

Q68 How did you learn about CBD? Choose all that apply

- TV (1)
- Newspaper (2)
- Friend or relative (3)
- JIA online support group (4)
- JIA blog (5)
- Radio (6)
- My own internet research (7)
- From by child's rheumatologist (8)
- From another physicain (9)
- Scientific journal article (10)
- Arthritis foundation (11)
- Other (12)

Q67 How much research have you done on CBD?

- A great deal (1)
- A lot (2)
- A moderate amount (3)
- A little (4)
- None at all (5)

Q69 What sources from your research did you find most credible? Choose all that apply

- ☐ TV (1)
- ☐ Newspaper (2)
- ☐ Radio (3)
- ☐ Friend or relative (4)
- ☐ Internet website (5)
- ☐ JIA online support group (6)
- ☐ JIA blog (7)
- ☐ Arthritis foundation (8)
- ☐ Scientific journal article (9)
- ☐ My child's rheumatologist (10)
- ☐ Another physican (11)
- ☐ Other (12)

Q71 How do you believe CBD will help your child's arthritis? Choose all that apply

- ☐ Reduce joint pain and inflammation (1)
- ☐ Keep or get arthritis into remission (2)
- ☐ Help reduce number of other medications prescribed (3)
- ☐ Help my child get off medications prescribed by their rheumatologist (4)
- ☐ Help reduce side effects of other medications (5)
- ☐ A natural therapy is effective for treating arthritis (6)
- ☐ I'm personally using CBD oil and found it to be helpful (7)
- ☐ Other (8)

Q90 If you selected "other" in the previous question, how else do you believe CBD will help your child's arthritis? (type in response)

____

____

____

____

____

Q72 Over the past week, how many days did you worry about the medications your child is using for treatment of their arthritis?

- ○ 0 (1)
- ○ 1 (2)
- ○ 2 (3)
- ○ 3 (4)
- ○ 4 (5)
- ○ 5 (6)
- ○ 6 (7)
- ○ 7 (8)

Q73 Over the past week, how many days did your child have side effects from the medications prescribed by their rheumatologist?

- ○ 0 (1)
- ○ 1 (2)
- ○ 2 (3)
- ○ 3 (4)
- ○ 4 (5)
- ○ 5 (6)
- ○ 6 (7)
- ○ 7 (8)

Q75 Do you think that there are side effects of CBD?

- yes (1)
- Maybe (2)
- No (3)

Q76 How safe or unsafe do you think CBD is?

- Very unsafe (1)
- Unsafe (2)
- Safe (3)
- Very safe (4)

Q77 Why do you think CBD is safe? Choose all that apply

- It's natural (1)
- It's available over the counter (2)
- Lots of research on CBD has been done (3)
- It's regulated by the Food and Drug Administration (FDA) (4)
- Friend or family member told me it was safe (5)
- Personal experience with CBD oil (6)
- Family of another child with JIA told me it was safe (7)
- Other (8)

Q78 If you selected "other' in the previous question, please type in other reasons why you think CBD is safe

____

Q81 How is your child's arthritis currently doing? (0= very poor, 10= very good). Move circle to number you've selected.

	0	1	2	3	4	5	6	7	8	9	10
Click to write Choice 1 ()											

Q82 Have you talked about CBD with your child's rheumatologist?

- Yes (1)
- No (2)

Q83 How did your child's rheumatologist react when you told him or her?

- Seemed upset (1)
- Neutral (2)
- Seemed interested (3)
- Seemed uninterested (4)
- Other (5)

Q84 If you selected "other" in the previous question, how else did your child's rheumatologist react when you told him or her about CBD.

____

Q85 Why have you not told your child's rheumatologist about possibly starting CBD? Choose all that apply

- ▢ Scared of what provider may think (1)
- ▢ Feel I won't be taken seriously (2)
- ▢ Haven't had the chance to talk to rheumatologist yet but planning to at next appointment (3)
- ▢ I don't think my child's rheumatologist will know anything about CBD oil (4)
- ▢ Other (5)

Q86 If you selected "other" in the previous question, please type in other reason(s) you haven't told your child's rheumatologist about CBD.

____

Q88 What type of CBD product are you considering using for your child? Select all that apply

- ▢ CBD oil balm (1)
- ▢ CBD oil spray (2)
- ▢ CBD drops (3)
- ▢ CBD soft gels or capsules (4)
- ▢ CBD gummies (5)
- ▢ CBD oil roll ons (6)
- ▢ Unsure yet (7)
- ▢ CBD tincture powder (8)
- ▢ CBD isolate (9)
- ▢ Other (10)

Q83 If you selected "other" in the previous question, please type in what other CBD product(s) you are considering to start for your child.

____

End of Block: Child demographics and CBD use
